# Seasonal Dynamics, Record of Ticks Infesting Humans, Wild and Domestic Animals and Molecular Phylogeny of *Rhipicephalus microplus* in Khyber Pakhtunkhwa Pakistan

**DOI:** 10.3389/fphys.2019.00793

**Published:** 2019-07-16

**Authors:** Abid Ali, Munsif Ali Khan, Hafsa Zahid, Pir Muhammad Yaseen, Muhammad Qayash Khan, Javed Nawab, Zia Ur Rehman, Muhammad Ateeq, Sardar Khan, Mohammad Ibrahim

**Affiliations:** ^1^Department of Zoology, Abdul Wali Khan University Mardan, Khyber Pakhtunkhwa, Pakistan; ^2^Department of Environmental Sciences, Abdul Wali Khan University Mardan, Khyber Pakhtunkhwa, Pakistan; ^3^Department of Microbiology, Abdul Wali Khan University Mardan, Khyber Pakhtunkhwa, Pakistan; ^4^Department of Chemistry, Abdul Wali Khan University Mardan, Khyber Pakhtunkhwa, Pakistan; ^5^Department of Environmental Sciences, University of Peshawar, Khyber Pakhtunkhwa, Pakistan

**Keywords:** ticks, hosts, *R. microplus*, Khyber Pakhtunkhwa, Pakistan

## Abstract

Although ticks prevalent in various agro-systems of Pakistan are associated with economic losses, information is still missing about the tick’s diversity, hosts they infest, seasonal dynamics and molecular phylogeny of *Rhipicephalus microplus* in Khyber Pakhtunkhwa (KP) Pakistan. This study for the first time enlisted ticks infesting diverse hosts including humans in various regions of KP. A total of 8,641 ticks were collected across the northern, southern and central regions of KP and were morpho-taxonomically categorized into six genera comprising 17 species, *R. microplus* (*n* = 3,584, 42%), *Hyalomma anatolicum* (*n* = 2,253, 27%)*, Argas persicus* (*n* = 1,342, 16%), *Hya. impeltatum* (*n* = 586, 7%)*, R. turanicus* (*n* = 161, 2%), *R. haemaphysaloides* (*n* = 142, 2%), *R. annulatus* (*n* = 132, 2%), *Hae. montgomeryi* (*n* = 123, 1.4%), *Hya. marginatum* (*n* = 110, 1.3%), *R. sanguineus* (*n* = 34, 0.4%), and *Hae. longicornis* (*n* = 31, 0.4%). Ticks infesting wild animals included *Amblyomma gervaisi*, *Amb. exornatum, Amb. latum, Dermacentor marginatus*, and *Hae. indica*, while ticks collected from humans included *R. microplus*, *R. annulatus, Hya. anatolicum, Hya. marginatum*, and *Hae. punctata*. The overall prevalence of ticks infesting domestic animals was 69.4% (536/772). Among animal hosts, cattle were found highly infested (87.2%, 157/180) followed by buffalos (79%, 91/114), domestic fowls (74.7%, 112/150), goats (68.3%, 82/120), dogs (66.7%, 32/48), horses (61.3%, 49/80), and sheep (16.3%, 13/80). Analysis revealed that the tick burden significantly differed among domestic animals and was found to be high in cattle, followed by buffalos, goats, sheep, domestic fowl, dogs, and horses. Seasonal patterns of ticks distribution showed highest prevalance in July, August, and September due to the prevailing high temperature and humidity during these months. The phylogenetic analysis of cattle tick *R. microplus* based on partial mitochondrial cytochrome oxidase subunit I (COX1), 16S ribosomal RNA (16S rRNA) and internal transcribed spacer 2 (ITS2) sequences, revealed that *R. microplus* prevalent in this region belongs to clade C which include ticks originating from Bangladesh, Malaysia, and India. Further large scale studies across the country are necessary to explore the molecular and cross breeding aspects at the geographical overlapping of various tick species and their associated pathogens to facilitate designing control strategies as well as awareness against tick infestation in the region.

## Introduction

Ticks are hematophagous arthropods, notorious vectors of human and animal pathogens and constitute anemerging economic and health problem in tropical and sub-tropical regions (de la Fuente et al., 2017). As a major challenge to public health and veterinary sector, ticks can harbor several pathogens that cause numerous infectious diseases. The emergence and resurgence of several tick-borne diseases pose increasing public health concerns (Gratz, 1999; Paddock and Childs, 2003; Jones et al., 2008). As a vector reservoir for several emerging and re-emerging infectious pathogens of medical and veterinary importance, ticks transmit a wide variety of arboviruses (Crimean-Congo hemorrhagic fever virus, tick-borne encephalitis virus), bacteria (*Rickettsia* spp.), spirochetes (*Anaplasma* spp. *Borrelia* spp. and *Ehrlichia* spp.) and protozoans (*Babesia* spp. and *Theileria* spp.), more than any other blood feeding arthropod (Labruna et al., 2004; Gondard et al., 2017; Schorderet-Weber et al., 2017). These voracious ectoparasites can infest wild, terrestrial, semi-aquatic vertebrates and humans, and are globally distributed from the Arctic to tropical regions (Sherrard-Smith et al., 2012; Yu et al., 2015). Ten percent of the total hard and soft tick species are known to transmit diseases to humans and other animals (Parola and Raoult, 2001; Lew-Tabor and Valle, 2016). Ticks and tick-borne diseases affect approximately 80% of the world’s cattle population, particularly in tropical and subtropical countries. Infestation with cattle tick *Rhipicephalus microplus* economically impact the livestock industry in different regions and annual losses due to only this tick have been estimated 22–30 billion US$ (Jabbar et al., 2015; Lew-Tabor and Valle, 2016).

The prevalence of ticks in different regions is mostly associated with various environmental factors that affect tick distribution and adaptation (Estrada-Peña, 2008). Like other hematophagous arthropods, ticks spend their life cycle in such an environment where they depend on host availability, host lifestyle, host interaction with other animals, vegetation coverage, habitat type and geo-climatic conditions (temperature and humidity) for their survival and development (Estrada-Peña, 2008; Gondard et al., 2017). Climatic changes influence tick distribution, shape the biodiversity of ticks and tick-borne pathogens and increase the risks of transmission of pathogens to humans and other hosts (Leger et al., 2013; Dantas-Torres, 2015). The dispersal of ticks in previously tick free areas is consociated with humans and other animals movements due to environmental changes (Estrada-Peña, 2008; Dantas-Torres, 2015) which has resulted in the emergence and re-emergence of tick-borne diseases.

Ticks that belong to the genus *Rhipicephalus* are responsible for severe economic losses in tropical and subtropical agro-systems. Previous studies have enlisted intraspecific variations in genus *Rhipicephalus* based on morpho-taxonomic complications in identification at species level (Lempereur et al., 2010; Barker and Walker, 2014). Morphological variations in *Rhipicephaline* ticks make it difficult to distinguish these tick species morphologically and to date, several valid species of *Rhipicephalus* (*Boophilus*) have been confirmed (Estrada-Peña et al., 2012; Guglielmone et al., 2014; Ali et al., 2016; Coimbra-Dores et al., 2018; Roy et al., 2018). The morpho-taxonomy of *R. microplus* (cattle tick) has been challenged in the last few years due to a hypothesis suggesting that biogeographical and ecological separations have occurred in Boophilid ticks across continents based on morphological and genetic variations (García-García et al., 1999; de la Fuente et al., 2000; Ali et al., 2016; Lew-Tabor et al., 2017). The genetic assemblage study based on mitogenome (12S rRNA and 16S rRNA) and microsatellites markers of *R. microplus* from America, Asia, Australia and Africa have confirmed that *R. microplus* consist of at least two different species (Labruna et al., 2009; Low et al., 2015; Ali et al., 2016; Baron et al., 2018). Mating experiments among these ticks evidenced that reproductive crosses between *R. microplus* ticks from Australia and Argentina or Mozambique are infertile while crosses between Boophilid ticks from Argentina and Mozambique are fertile. These findings based on genetic divergence and reproductive isolation put forward that the Australian *R. microplus* strain is different from the American, Asian and African strain. Phylogenetic analysis of the *R. microplus* mitogenome (COX1 and 16S rRNA) and internal transcribed spacer (ITS) gene revealed that *R. microplus* is a complex of species. The cryptic diversity of *R. microplus* complex includes *R. annulatus*, *R. australis* and two clades of *R. microplus*, clade A and B. Clade A *R. microplus* includes ticks from America and Africa and clade B includes ticks from China (Labruna et al., 2009; Burger et al., 2014; Low et al., 2015; Roy et al., 2018). Recent mitogenome approaches based on COX1 and 16S rRNA haplotypes suggested a distinct genetic assemblage of *R. microplus* from Malaysia resulting in novel clade C which includes ticks from Pakistan, Bangladesh, Myanmar, India and Malaysia (Low et al., 2015; Baron et al., 2018; Roy et al., 2018).

In Pakistan, valid studies have shown the prevalence of five tick genera, including *Hyalomma*, *Haemaphysalis*, *Rhipicephalus, Ornithodoros*, and *Argus* (Karim et al., 2017; Rehman et al., 2017). The notable species compositions of genus *Hyalomma* are *Hya. anatolicum*, *Hya. scupense*, *Hya. kumari*, *Hya. isacci, Hya. turanicum* and *Hya. dromedarii*; genus *Haemaphysalis* are *Hae. bispinosa*, *Hae. montgomeryi*, *Hae. cornupunctata*, *Hae. kashmirensis*, and *Hae. sulcata*; genus *Rhipicephalus* species compositions include *R. microplus, R. annulatus, R. haemaphysaloides, R. turanicus*, and *R. sanguineus;* the genus *Argus* is composed of *Argas persicus* and genus *Ornithodoros* is represented by *Ornithodoros tolozani* (Hoogstraal and Varma, 1962; Kaiser and Hoogstraal, 1964; Robertson et al., 1970; Karim et al., 2017; Rehman et al., 2017).

Pakistan is an agricultural country where livestock plays a crucialrole in the national economy. In Pakistan, climatic conditions are extremely favorable for the growth and survival of diverse tick species. These ticks cause severe problems for livestock holders which mainly include low income farmers. The majority of these farmers consider all tick species as one species and are mostly unaware of the capability of pathogens transmission to humans as well as other associated health risks. Tick and tick-borne diseases are prevalent in the province Khyber Pakhtunkhwa (KP) of Pakistan, causing economic loss to livestock holders (Jabbar et al., 2015; Karim et al., 2017; Rehman et al., 2017). However, there is a paucity of documented information about ticks spatial distribution and diversity, their diverse hosts and seasonal dynamics from this region. Therefore, the present endeavor was focused on tick diversity to investigate the present status of tick species infesting various animal hosts, their seasonal dynamics and to obtain the evolutionary information of cattle tick *R. microplus* which is prevalent in the study area.

## Materials and Methods

### Study Area

**FIGURE 1:**
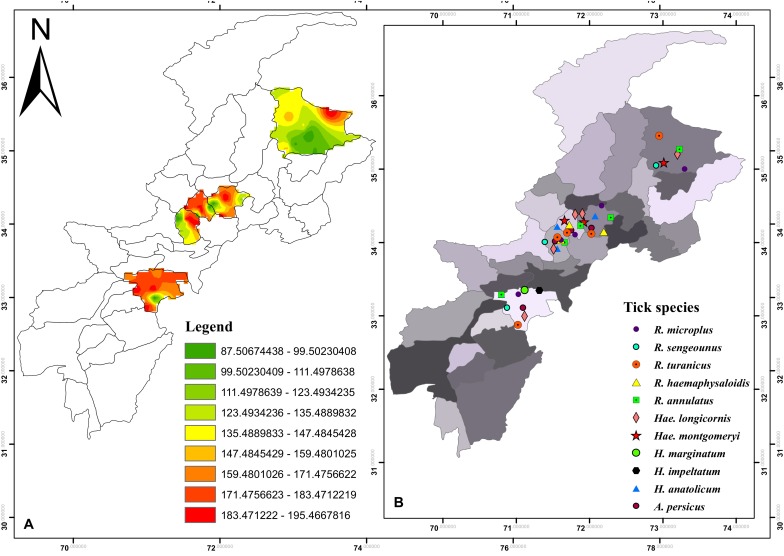
**(A)** Density map of tick burden in the selected districts **(B)** and spatial distribution of tick species in KP.

During the present study five districts namely Charsadda (34°09′49.4′′N 71°44′53.4′′E), Mardan (34°11′54.6′′N 72°01′37.4′′E), Peshawar (34°01′36.2′′N 71°31′47.4′′E), Lower Kohistan (35°19′48.2′′N 73°13′50.7′′E), and Karak (33°06′37.5′′N 71°05′29.0′′E), in the northern, southern and central KP (Northwestern geographic state of Pakistan previously known as North-West Frontier Province) were selected to assess the diversity of ticks, their hosts and seasonal dynamics. Nine regions of each aforementioned districts were selected for ticks sampling (Figure 1). A Global Positioning System (GPS) was used to obtain geographic coordinates data and loaded onto a Microsoft Excel sheet to develop a distribution map for the study areas using ArcGIS 10.3.1.

### Ethics Statement

The protocol for the present study was approved by the ethical committee and board of study members of the Department of Zoology Abdul Wali Khan University Mardan. Written informed consent was obtained from the owners of animals and individuals infested by ticks prior to tick collection.

### Ticks Collection

Tick samples were collected during April 2017 to March 2018, from different domestic animals (cattle, buffalo, horse, sheep, goat, dogs, and domestic fowl) at different localities (villages and towns) in various tehsils (an administrative division within the district) of the aforementioned districts (Figure 1). For ticks collection, a regular visit was made three times a month and sampling was done randomly with the help of fine tweezers from sampling animals reared at animal farms, houses or grazing in the field. Collected specimens were rinsed with distilled water followed by absolute ethanol to remove any surface confined microbes and host contaminating tissues and stored in properly labeled separate plastic tubes until further analyses. The relevant information including age of host, physical status, previous acaricides or any drugs used against ticks, the body region from where ticks were isolated, date and place of collection were recorded and all the samples were shipped to the Parasitology laboratory of the department of Zoology, Abdul Wali Khan University Mardan. Ticks were immediately identified or preserved (100% ethanol or in a mixture of 95% ethanol, 4% distilled water and 1% glycerol) (Walker et al., 2003) for further study.

**FIGURE 2:**
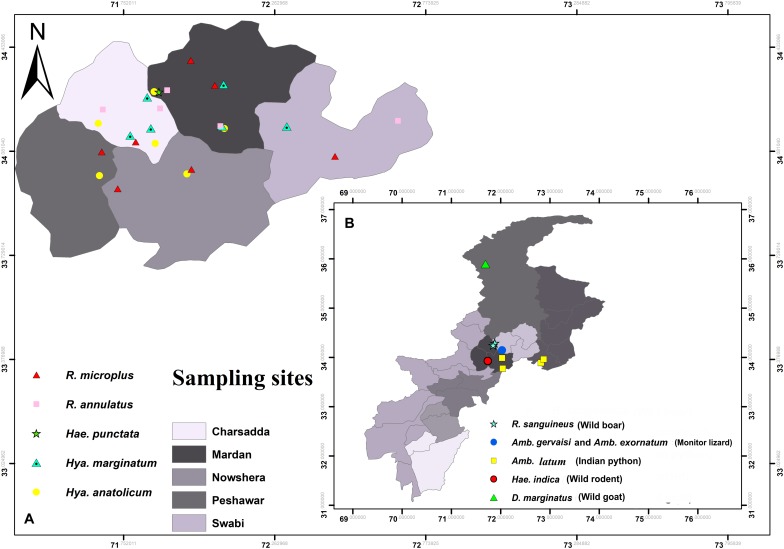
Map showing locations of ticks **(A)** collected from humans **(B)** and wild animals.

During collection, ticks were accidentally found infesting humans and wild goats (locally known as markhor, a national animal of Pakistan mostly found on the Himalaya Mountains). Wild rodents (Indian gray mongoose), wild boars (wild pig), and wild reptiles (monitor lizard and Indian python) were captured by local farmers or found dead on highways. In this collection, ticks were also included from districts other than the above-mentioned districts such as Chitral (35°53′40.9′′N 71°41′31.1′′E), Haripur (33°59′41.0′′N 72°54′35.3′′E), Swabi (34°07′29.1′′N 72°27′25.7′′E), and Nowshera (34°00′27.8′′N 71°59′09.8′′E) (Figure 2).

### Morpho-Taxonomic Identification of Ticks

Tick samples were identified morphologically using tick morpho-taxonomic characters under stereozoom microscope (HT Stereozoom) according to the standard relevant identification keys (Walker et al., 2003; Apanaskevich and Horak, 2005, 2008, 2009; Ghosh and Misra, 2012; Nowak-Chmura, 2012; Madder et al., 2014; Caetano et al., 2017).

### DNA Extraction and Molecular Identification

A total of 225 (45 per district) morphologically identified *R. microplus* from nine locations in each of the five districts were selected for genomic DNA extraction. Tick samples from each district were separately pooled into five groups, each pool containing ticks of a different location within the district. Ticks were cleaned with distilled water, dried and cut into small pieces using a sterile scalpel and homogenized by a sterile micro pestle in separate 1.5 ml Eppendorf tubes. Genomic DNA was separately extracted from each pool of tick samples using the GeneJET Genomic DNA Purification Kit (Thermo Fisher Scientific) according to the standard DNA extraction protocol of the manufacturer. DNA concentration in the samples was quantified using a Nanodrop ND-100 (Thermo Fisher Scientific) and stored at −20°C until further processing. COX1, 16S rRNA and ITS2 gene fragments of *R. microplus* were amplified by PCR in a thermocycler (HT, ILF, UK) using specific primers (Table 1). PCR reactions were performed in a total volume of 25 μL reaction mixture with a composition of 1 μL of each forward and reverse primer, 2 μL of template DNA (50 ng), 7.5 μL of deionized water and 13.5 μL of master mix (Dream*Taq* PCR Master Mix [2X] (Thermo Fisher Scientific). The thermocycling conditions for COX1, 16S rRNA and ITS2 were optimized separately for each reaction. Standardized PCR conditions was, an initial denaturation of 5 min at 95°C followed by 35 cycles of denaturation at 95°C for 30 s, annealing at 53.8°C for COX1, 55.9°C for 16S rRNA and 58°C for ITS2 for 30 s, an extension at 72°C for 30 s and a final extension of 10 min at 72°C. A negative control (distilled water) was included in each run of the amplification reaction for validation. PCR products were confirmed by running the amplified DNA in 2% ethidium bromide stained agarose gel with a 50 bp DNA marker (Thermo Fisher Scientific). The results were visualized using the GeneDoc (UVP BioDoc-It Imaging System).

**TABLE 1:** Primers used for the amplification of target partial genes of *R. microplus.*

**Gene**	**Primer sequence**	**Amplicons size (nt)**	**References**
COXI	F: ATTTTACCGCGATGAATATACTCTAC	620 bp	Present study
	R: TCTGTTAATAGTATGGTAATAGCACCTG		
16S rRNA	F: ATTTTGACTATACAAAGGTATTGAAAT	376 bp	Present study
	R: ATTTAAAAGTTGAACAAACTTCTTATTT		
ITS2	F: CACATATCAAGAGAGCCTTCGGC	267 bp	Present study
	R: CATCGTCTTGTGTAGCGTCGC		

### DNA Purification and Sequencing

The amplified PCR products were purified using the GeneClean II kit (Qbiogene) according to the manufacturer’s instructions. For the sequencing of PCR based amplified product, 45 DNA samples of each district were separately grouped containing fifteen samples for each gene fragment. The purified PCR amplicons (45 PCR product samples, 15 samples for each marker) were sequenced unidirectionally by Macrogen Inc. (Seoul, South Korea). The obtained sequences (nucleotide sequences) were analyzed using BioEdit V. 7.0.5 (Hall et al., 2011) and NCBI BLAST^1^ (Altschul et al., 1990). All the relevant library sequences of closely related species and of *R. microplus* complex available in GenBank were downloaded and saved for downstream analysis to construct a phylogenetic tree. The obtained sequences were trimmed for the removal of unnecessary nucleotides and those with 100% similarity with each other were discarded. Finally, 12 sequences were uploaded to NCBI GenBank including COX1 (4), 16S rRNA (3) and ITS2 (5).

### Phylogenetic Analysis

**FIGURE 3:**
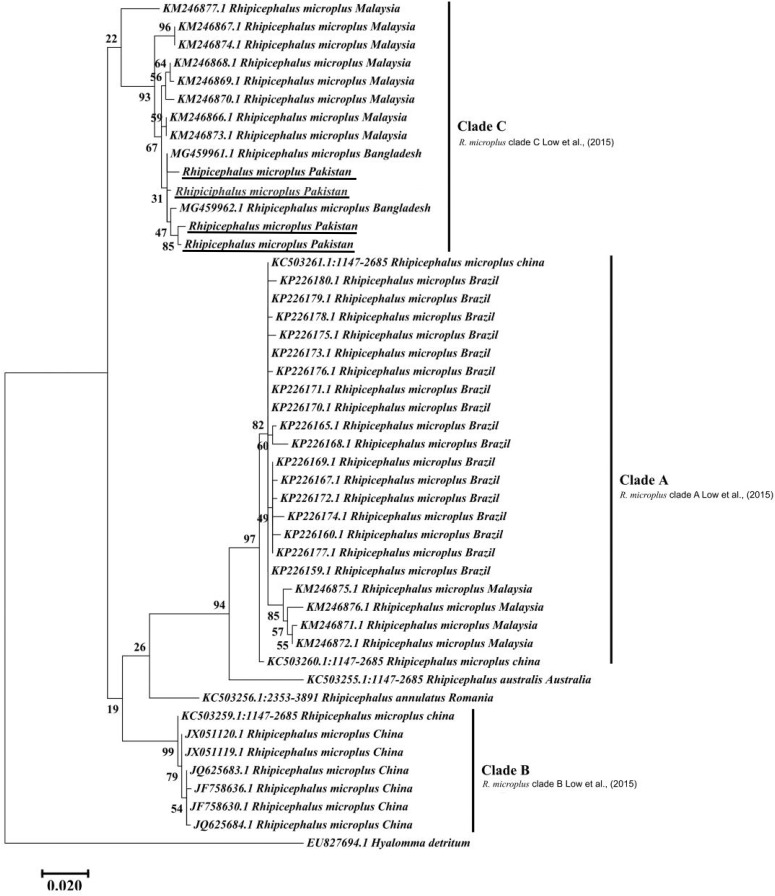
Maximum likelihood tree inferred from COX1 sequences of the genus *Rhipicephalus* and using *Hyalomma* sequence as outgrowth. GenBank accession numbers are followed by species name and location of collection. Clade A includes *R. microplus* ticks from America, Malaysia, and China, clade B includes tick from China and clade C includes ticks from Pakistan, India, Bangladesh, Myanmar, and Malaysia. Support values (Bootstrapping values) were indicated at each node. The bar represents 0.020 substitutions per site. Sequences obtained in the present study were underlined.

**FIGURE 4:**
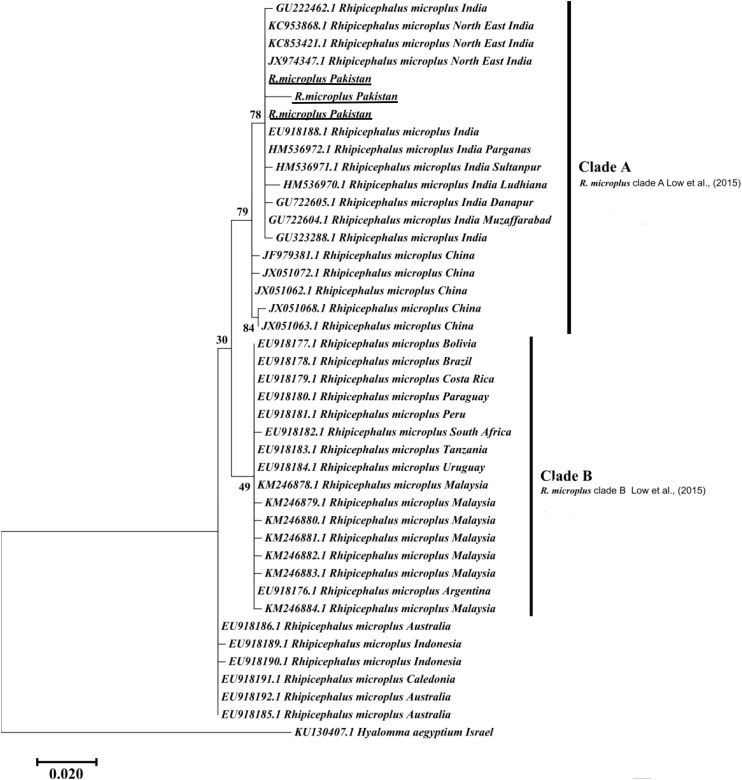
Maximum likelihood tree inferred from 16S rRNA sequences of the genus *Rhipicephalus* and using *Hyalomma* sequence as outgrowth. GenBank accession numbers are followed by species name and location of collection. The clade A includes *R. microplus* from Pakistan, India, and China while Clade B contains *R. microplus* from Africa, Malaysia and South America. Support values (Bootstrapping values) were indicated at each node. The bar represents 0.020 substitutions per site. Sequences obtained in the present study were underlined.

**FIGURE 5:**
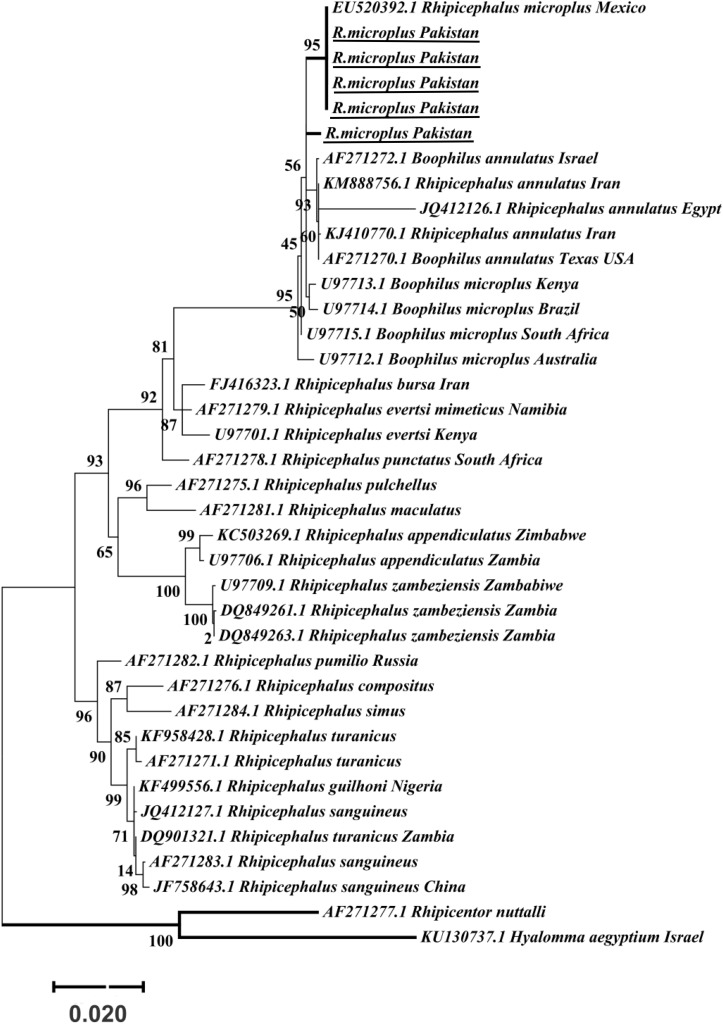
Maximum likelihood tree inferred from ITS2 sequences of the genus *Rhipicephalus* and using *Rhipicentor* and *Hyalomma* sequences as outgrowth. GenBank accession numbers are followed by species name and collection location. Support values (Bootstrapping values) were indicated at each node. The bar represents 0.020 substitutions per site. Sequences obtained in the present study were underlined.

The obtained trimmed sequences were aligned using ClustalW in BioEdit Sequence Alignment Editor V 7.0.5 (Hall et al., 2011). To test molecular phylogeny of the cattle tick *R. microplus* a multi locus analysis was performed for COX1 (Figure 3), 16S rRNA (Figure 4), and ITS2 (Figure 5) partial sequences using the Maximum likelihood method in MEGA v. X (Kumar et al., 2018) with bootstrapping at 1000 replications (Tamura et al., 2013).

### Statistical Analysis

The overall tick prevalence was calculated by the total ticks divided by total hosts, host wise infestation was calculated by the infested hosts divided by total examined. The mean percent of collected ticks and binomial confidence intervals for proportion were calculated (Table 2). The median tick burden was analyzed on hosts by applying the Kruskal–Wallis test (*P* < 0.001) among different hosts and the Pearson correlation test was applied for the temporal distribution of ticks. The results were considered statistically significant at *p* < 0.05.

**TABLE 2:** Tick species collected from diverse hosts across KP, Pakistan.

**Host animal**	***Hyalomma***			***Rhipicephalus***					***Haemaphysalis***		***Argus***
	*Hya. anatolicum* L/M/F^*^	***Hya. marginatum* L/M/F**	***Hya. impeltatum* L/M/F**	***R. microplus* L/M/F**	***R. haemaphysaloides* L/M/F**	***R. sanguineus* L/M/F**	***R. turanicus* L/M/F**	***R. annulatus* L/M/F**	***Hae. montgomeryi* L/M/F**	***Hae. longicornis* L/M/F**	***A. persicus* L/M/F**
Cow	207/976/561	4/31/23	34/91/161	148/353/1291	8/13/40	1/2/8	10/17/34	19/27/33	0/26/21	0/3/6	–
Buffalo	41/89/39	0/9/12	8/40/87	13/29/182	–	–	3/16/19	–	–	–	–
Goat	11/54/22	2/10/5	0/25/28	7/184/398	4/16/22	–	9/0/2	0/23/14	4/0/8	–	–
Sheep	13/39/53	0/0/3	0/8/13	23/137/296	0/9/18	–	2/13/13	0/11/5	14/17/12	1/2/5	–
Dog	–	–	–	–	–	7/7/9	3/0/14	–	–	7/2/2	–
Horse	19/94/35	0/8/3	12/35/44	61/196/266	0/4/8	–	0/4/2	–	3/4/14	0/0/3	–
Domestic fowl	–	–	–	–	–	–	–	–	–	–	178/317/847
Total	2,253	110	586	3584	142	34	161	132	123	31	1,342
Mean (%)	26.80	1.38	6.88	41.90	1.68	0.44	1.89	1.55	1.48	0.40	15.60
95% CI	(25.8–28.0)	(1.0–2.56)	(5.3–8.0)	(40.2–43.7)	(1.0–2.8)	(0.1–1.12)	(1.1–3.56)	(1.7–2.20)	(1.0–2.31)	(0.1–1.20)	(14.1–17.3)

^*^Nymphs or partially fed ticks were considered as adults (F). L, larvae; M, male. CI, Confidence Interval.

## Results

### Tick’s Description and Host Population

**FIGURE 6:**
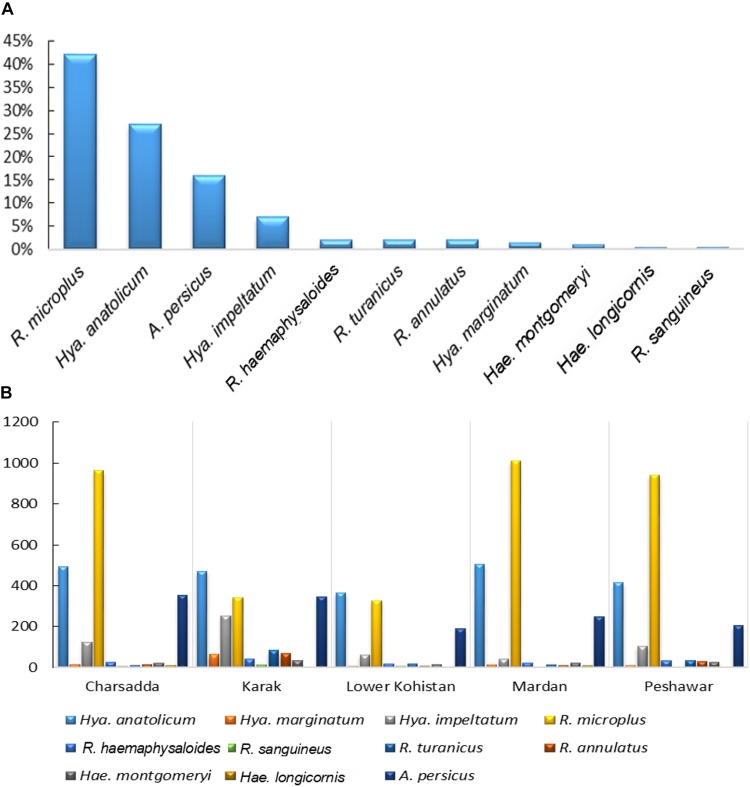
**(A)** shows percent composition of collected tick species **(B)** and spatial distribution of ticks in various districts of KP, Pakistan.

Morpho-taxonomic analysis categorized collected ticks into six genera including 17 species (Figure 1). The highest number of collected tick species was *R. microplus* (*n* = 3584, 42%) followed by *Hya. anatolicum* (*n* = 2,253, 27%)*, A. persicus* (*n* = 1,342, 16%), *Hya. impeltatum* (*n* = 586, 7%)*, R. turanicus* (*n* = 161, 2%), *R. haemaphysaloides* (*n* = 142, 2%), *R. annulatus* (*n* = 132, 2%), *Hae. montgomeryi* (*n* = 123, 1.4%), *Hya. marginatum* (*n* = 110, 1.3%), *R. sanguineus* (*n* = 34, 0.4%), and *Hae. longicornis* (*n* = 31, 0.4%) (Table 2 and Figure 6A). Cattle were found infested separately by *R. microplus, R. haemaphysaloides, Hya. anatolicum, Hya. impeltatum, Hya. marginatum* and *Hae. montgomeryi* and buffalos were found infested separately by *R. microplus, R. haemaphysaloides*, *Hya. anatolicum*, and *Hae. montgomeryi*. On the other hand, goats were found infested by *Hae. montgomeryi, Hae. longicornis*, and *Hya. impeltatum*, and sheep were infested separately by two species *Hae. longicornis* and *Hya. impeltatum. R. annulatus* and *R. sanguineus* were collected from dogs. Horses and domestic fowls were found to be infested by a single species of *R. turanicus* and *A. persicus*, respectively. Hosts infested by multiple tick species were also recorded. For instance, *R. microplus* and *Hya. anatolicum* were found infesting both cattle and buffaloes (Table 3).

**TABLE 3:** Collected ticks, their hosts and preferred attachment sites on different hosts observed during present study.

**Host**	**Tick species collected**	**Tick attachment site on host body**
Cattle	*R. microplus, R. haemaphysaloides, Hya. anatolicum, Hya. impeltatum, Hya. marginatum*, and *Hae. montgomeryi*	(belly, dewlap, shoulders and flanks) (axillae, groin, genital areas, perineum and udder)
Buffalos	*R. microplus R. haemaphysaloides*, *Hya. anatolicum* and *Hae. montgomeryi*	(neck, shoulders, flanks, axillae, groin, genital areas, perineum, and udder)
Goat	*Hae. longicornis, R. haemaphysaloides* and *Hya. impeltatum*	(neck, shoulder, groin, axillae, genital areas, perineum, and udder)
Dog	*R. annulatus* and *R. sanguineus*	(legs, shoulders, ears, and neck)
Sheep	*Hae. longicornis* and *Hya. impeltatum*	(groin, axilla, lags, genital areas perineum, and udder)
Horses	*R. turanicus*	(shoulder)
Domestic fowl	*A. persicus*	(host plumage and nests)

**Multiple tick species infesting same host**		

Cattle		*R. microplus* and *Hya. anatolicum*
Buffalos		*R. microplus* and *Hya. anatolicum*

A total of 772 animals were observed throughout the study period (April 2017 to March 2018) which included bovine (cattle, 180; buffalos, 114), ovine (sheep, 80), caprine (goats, 120), avian (domestic fowl, 150) equine (horse, 80) and carnivorous (dogs, 48) hosts, from which 8,498 ticks were collected. The overall prevalence of tick infested animals was 69.4% (536/772). Among the tick infested hosts; cattle were found highly infested (87.2%, 157/180) followed by buffalos (79.8%, 91/114), domestic fowl (74.7%, 112/150), goats (68.3%, 82/120), dogs (66.7%, 32/48), horses (61.3%, 49/80), and sheep (16.3%, 13/80).

### Tick Burden

Median tick burden recorded on host animals was 16 (median 16, Q1-Q3: 9-31) and showed a statistically significant difference among the different host groups (χ^2^ = 157.83, *df* = 6, *p* < 0.001). Median tick burden was high in cattle (median 29), followed by buffalos (median 21.5), goats (median 21), sheep (13), domestic fowl (12), dogs (8.5), and horses (6).

### Seasonal Dynamics of Ticks

The tick species included in the seasonal dynamics analysis were; *Hya. anatolicum, Hya. marginatum, Hya. impeltatum, R. microplus, R. haemaphysaloides, R. sanguineus, R. turanicus, R. annulatus, Hae. montgomeryi, Hae. longicornis*, and *A. persicus.* Tick species showed spatio-temporal variations in their distribution. Specifically, species of the genus *Hyalomma* were mainly reported in dry regions while species belonging to the genus *Rhipicephalus* were reported in semi-arid regions of the study area (Figure 6B). During ticks collection, adult females (partially fed or nymphs) were highly prevalent followed by males and larvae (Table 2). Other tick species were not included in the temporal analysis because they were collected by chance from novel hosts (these hosts were not the focus of the present study) either from the study area or from other districts within KP Pakistan, during some parts of the year. This included tick species such as *Amb. gervaisi, Amb. exornatum, Amb. latum, D. marginatus, Hae. indica*, and *R. sanguineus* from monitor lizards, Indian pythons, markhor, mongoose and wild boars, respectively.

**FIGURE 7:**
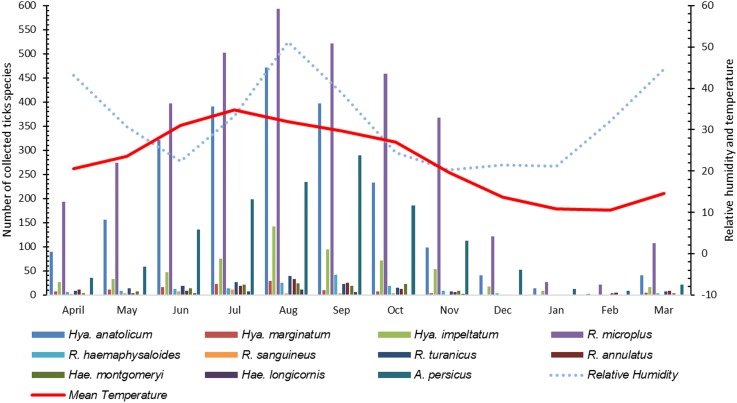
Seasonal dynamics of various tick species recorded during this study.

The highest number of ticks were collected in July followed by August and September as an increase in the infestation rate of ticks were recorded during ticks collection and these finding significantly correlated with an increase in mean temperature [*r*(10) = 0.929, *p* < 0.01] and relative humidity [*r*(10) = 0.831, *p* < 0.01] during these months in the region. As the mean temperature fell and relative humidity decreased, the infestation rate also decreased (Figure 7).

### Ticks Infesting Humans and Wild Animals

**FIGURE 8:**
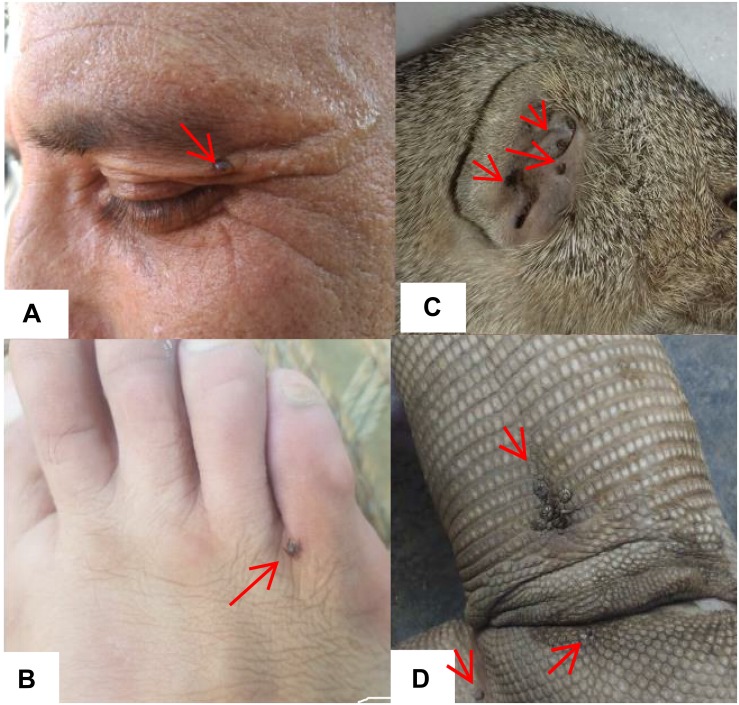
Figure showing ticks infesting humans **(A,B)** (written informed consent was obtained from the individuals for the publication of images), *Hae. indica* collected from mongoose (*Herpestes edwardsi*) **(C)**, and *Amb. gervaisi* collected from monitor lizards (*Varanus varanus*) **(D)**.

A total of 25 ticks were collected from humans (i.e., three genera and five species including *R. microplus, R. annulatus, Hae. punctata, Hya. marginatum*, and *Hya. anatolicum*). Ticks collected from wild animals included *Hae. indica* (wild rodent, *n* = 17), *Amb. gervaisi* and *Amb. exornatum* (monitor lizard, *n* = 21 and *n* = 14), *Amb. latum* (Indian python, *n* = 23), *D. marginatus* (wild goat, *n* = 31) and *R. sanguineus* (wild boar, *n* = 12) (Table 4 and Figure 8).

**TABLE 4:** Tick species collected from wild animals during this study.

**Infected host**	**Collection date**	**Collected tick species**	Number and life stage^*^
Wild rodent (*Herpestes edwardsi*)	March 17, 2018	*Hae. indica*	17 (4L,10M,3F)
Monitor lizard (*Varanus varanus*)	June 12, 2018	*Amb. gervaisi*	21 (3L,7M,11F)
Monitor lizard (*Varanus varanus*)	July 15, 2018	*Amb. exornatum*	14 (2L,5M,7F)
Wild goat (*Capra falconeri*)	August 21, 2017	*D. marginatus*	31 (5L,9M,17F)
Wild boar (*Sus scrofa*)	May 05, 2017	*R. sanguineus*	12 (2L,6M,4F)
Indian python (*Python molurus*)	July 10, 2018	*Amb. latum*	23 (3L,9M,11F)

^*^Nymphs or partially fed ticks were considered as adults (F). L, larvae; M, male.

### Sequence and Phylogenetic Analysis

Genomic DNA extracted from morphologically identified *R. microplus* tick samples were amplified by PCR using COX1, 16S rRNA and ITS2 specific primers (Table 1). Results were observed on agarose gel electrophoresis and the resultant amplicons (COX1, 16S rRNA and ITS2) were 620, 376, and 267 bp, respectively. After sequencing, BLAST analysis of the COX1 nucleotide partial sequences showed 95–100% identity with the same sequence reported from Pakistan (accession no. MG459963), Bangladesh (accession no. MG459961, MG459962) Myanmar (accession no. MG459964) and China (accession no. KC503259). In the case of 16S rRNA nucleotide partial sequences, BLAST analysis revealed that it shares a 98–100% identity with previously reported sequences for *R. microplus* from India (accession no. KY458969; MG811555) and China (accession no. KU664521; MH208600). ITS2 partial nucleotide sequences showed that it shares 99% identity with the same sequences reported for the same tick species from India (accession no. JX974346; MG978179), China (accession no. JQ737125; JQ737124; KX450290; MG721034), Laos (accession no. KC503276), Colombia (accession no. MF353138), and Cambodia (accession no. KC503272). Similarly, the sequences of COX1 (accession no. MK812968, MK836289, MK858219, MK858220), 16S rRNA (accession no. MK463980, MK463981, MK463982) and ITS 2 (accession no. MK480725, MK524212, MK531135, MK578158, MK577644) obtained in the present study were deposited to the NCBI databank.

Maximum likelihood (ML) trees were inferred from COX1, 16S rRNA and ITS2 partial sequences of *R. microplus* to establish its phylogenetic relationship. ML analysis of COX1 nucleotide sequences revealed three clades, clade A includes ticks from America, Malaysia, and China, clade B was comprised of ticks originating from China (Burger et al., 2014; Low et al., 2015) and clade C includes ticks from Pakistan, India, Bangladesh, Myanmar, and Malaysia (Figure 3) (Roy et al., 2018). The COX1 phylogenetic analysis provided better support to resolve the evolutionary relationships of *R. microplus*. On the other hand, 16S rRNA partial sequences provided little phylogenetic structure of *R. microplus* complex and divided into two phylogenetic clades, the clade A comprised of the *R. microplus* tick species from China, India and Pakistan. Clade B includes *R. microplus* ticks originating from Malaysia, Africa and America and the *R. australis* (formerly recognized as *R. microplus*) from Australia, Indonesia and New Caledonia formed a separate subclade along with the aforementioned ticks (Figure 4). Based on ITS2 analysis, all species of the *R. microplus* complex were clustered together and the ITS2 tree provided support to the monophyly of *R. microplus* complex (Figure 5).

## Discussion

Climatic change and global warming have a vital impact on the distribution of ticks and tick-borne pathogens, as each tick species selects a set of ecological conditions and biotopes that determine its dispersal and outline risk areas for their associated pathogens transmission (Leger et al., 2013). Tick habitat expansion and novel pathogens (protozoans, bacteria, rickettsia, and viruses) are re-emerging in new geographic areas, posing a serious threat to public and veterinary health (Estrada-Peña et al., 2013; Jore et al., 2014). In KP Pakistan, the majority of farmers lack basic knowledge on ticks, their disease causing potential and the wide variety of hosts they infest, mostly resulting in a failure of controlling tick infestations. The present study encompasses the identification of diverse tick species and novel hosts, including humans, along with the seasonal dynamics and molecular phylogeny of *R. microplus* from north-western regions of Pakistan. The collected ticks were categorized into six genera including 17 species of medical and veterinary concern. We reported *Amb. latum*, *Amb. gervaisi*, *Amb. exornatum, A. persicus*, *D. marginatus*, *Hae. indica*, *Hae. longicornis*, *Hae. punctata, Hae. montgomeryi, Hya. impeltatum, H. anatolicum, Hya. marginatum*, *R. annulatus, R. haemaphysaloides*, *R. microplus*, *R. sanguineus*, and *R. turanicus* from the KP province. The majority of these ticks are responsible for the transmission of *Babesia bovis, Theileria annulata*, *Anaplasma marginale*, and *Anaplasma centrale* in the region (Jabbar et al., 2015). Our findings on the tick species prevalent in Pakistan correspond with previously reported studies (Manan et al., 2007; Karim et al., 2017; Rehman et al., 2017). Additionally, these ticks have been shown to be responsible for considerable losses in the livestock industry, causing severe threats to animal hosts and public health (Tadesse et al., 2012; Rehman et al., 2017).

Although ticks infestation on humans has been recorded from other regions of the world (Guglielmone and Robbins, 2018), ticks infesting humans and wild animals have not been previously reported in KP, Pakistan. During this study we found *R. microplus*, *R. annulatus, Hya. anatolicum, Hya. marginatum* and *Hae. punctata* (three genera and five species) infesting humans (Figure 8). These ticks, for instance, *R. microplus* (Labruna et al., 2005; Okino et al., 2010; Serra-Freire, 2010), *R. annulatus* (Horak et al., 2005; Bakirci et al., 2014; Kar et al., 2017) *Hya. anatolicum* (Estrada-Peña and Jongejan, 1999; Apanaskevich and Horak, 2005; Papa et al., 2011), *Hya. marginatum* (Santos-Silva et al., 2011), and *Hae. punctata* (Fernández-Soto et al., 2006; Briciu et al., 2011) parasitizing humans have already been reported in other parts of the world. *Hae. indica* from Indian gray mongoose are reported in this study, tick infestation on mongoose have been reported previously (Hoogstraal, 1970; Hoogstraal and Wassef, 1977; Horak et al., 1999; Cheng et al., 2018). In the present study *Amb. gervaisi* and *Amb. exornatum* parasitizing monitor lizards, and *Amb. latum* infesting Indian pythons were also reported. *Amblyomma* ticks are vectors of several pathogens known to transmit disease causing agents to humans and has never been investigated in Pakistan. *Amblyomma* species infesting reptiles are vectors of *Aeromonas hydrophila* that cause bacterial stomatitis, paralysis and pneumonia in snakes (Tenderio, 1953; Stephen and Achyutha, 1979; Marcus, 1981; Stenos et al., 2005; Hanson et al., 2007). *D. marginatus* were found to infest wild goats (locally known as markhor), which demands further studies to investigate the negative impact of this tick species in markhor which is considered as endangered species. The genus *Dermacentor* is of public and veterinary health concern as a vector reservoir for several pathogens with unknown potential risks in Pakistan. *R. sanguineus*, a vector of microorganisms of medical and veterinary health concern such as *Hepatozoon canis* and *Ehrlichia canis* (Dantas-Torres et al., 2018; Cabezas-Cruz et al., 2019) was collected for the first time from wild boars in the region.

Studies showing the seasonal pattern of tick distributions have never been reported from KP, Pakistan. During this study, information about the tick burden associated with relative humidity and temperature was recorded which is essential for the timing of potential control measures in tick infestation season. The target study region has high temperature and humidity during July, August and September therefore, high tick infestations were recorded during these months. On the other hand, due to the low temperatures during December, January, and February, several tick species were found to exhibit low infestation rates.

Several studies have used COX1, 16S rRNA and ITS2 as genetic markers for the accurate molecular identification and phylogenetic relationship of various organisms, including hard ticks such as *R. microplus* (Burger et al., 2014; Lv et al., 2014; Low et al., 2015; Coimbra-Dores et al., 2018). COX1 sequences have been used to show that cryptic species of *R. microplus* contain and display different population structures in different geographical regions (Burger et al., 2014; Low et al., 2015). Thus, *R. microplus* appears to consist of a complex of distinct genetic assemblages, namely *R. australis*, *R. annulatus*, *R. microplus* clade A (Burger et al., 2014), *R. microplus* clade B (Burger et al., 2014), and *R. microplus* clade C (Low et al., 2015). Burger et al. (2014) showed that *R*. *microplus* from clade B (Southern China and Northern India) is more closely related to *R*. *annulatus* than to *R*. *microplus* from clade A (Asia, South America, and Africa). In accordance with previous studies, phylogenetic trees based on COX1, 16S rRNA and ITS2 of *R. microplus* were obtained to establish the phylogenetic relationship of this tick (Lv et al., 2014; Low et al., 2015; Csordas et al., 2016; Rehman et al., 2017; Roy et al., 2018). COX1 was found to be an established marker for the phylogenetic analyses and informative for *R. microplus* phylogeny as compared to 16S rRNA and ITS2 as the latter provides monophyletic support to Boophild ticks (Burger et al., 2014). The COX1 phylogenetic analysis provided support to resolve the evolutionary relationship of *R. microplus*. Our findings about obtained COX1 sequences and phylogenetic analysis are parallel with previous reports from the region (Roy et al., 2018). ML analysis of COX1 nucleotide sequences revealed that these ticks are closely related to clade C (Low et al., 2015; Roy et al., 2018) (Figure 3). On the other hand, 16S rRNA partial sequences provided support to the monophyly of *R. microplus* complex and our tick sequences were clustered with clade A (Figure 4) (Brahma et al., 2014; Lv et al., 2014; Low et al., 2015). Based on ITS2 analysis, all species of the *R. microplus* complex were clustered together on the tree and provided support to the monophyly of *R. microplus* complex. The phylogenetic relationship in the *R. microplus* species complex was poor and could not accurately categorized as a sister species since ITS2 is highly conserved and is insufficient in distinguishing these closely related species (Figure 5).

## Conclusion

This study for the first time explored the tick diversity infesting various hosts in KP, Pakistan. The important tick species of domestic animal hosts are *R. microplus, R. annulatus, R. haemaphysaloides, R. sanguineus, R. turanicus, Hae. montgomeryi, Hae. longicornis, Hya. marginatum. Hya. anatolicum, Hya. impeltatum*, and *A. persicus.* The seasonal pattern of several tick species showed high infestation during July, August and September in which high temperature and humidity persists in the region. The collection of tick species parasitizing humans in the study area may facilitate further studies to identify the functional role of these parasites in the transmission of pathogens that cause human diseases. Altogether, tick species such as *Hae. indica*, *Amb. gervaisi*, *Amb. exornatum, Amb. latum*, *D. marginatus*, and *R. sanguineus* were reported for the first time infesting wild animals in the region. The phylogenetic relationship of cattle tick *R. microplus* explored in this study will further help in defining control strategies. These findings will facilitate awareness among the local population about tick species diversity and their hosts and future control strategies against ticks and tick-borne diseases in Pakistan.

## Data Availability

The datasets generated for this study can be found in NCBI, MK463980, MK463981, MK463982, MK480725, MK524212, MK531135, MK578158, MK577644, MK812968, MK836289, MK858219, MK858220.

## Author Contributions

AA designed the study and acquired the budget. AA, MAK, HZ, SK, PY, JN, ZUR, MA, and MI collected the samples. AA, HZ, SK, MQK, MAK, and JN carried out the statistical analysis. MAK, HZ, PY, ZUR, AA, MA, and MI conducted the experiments. All authors carried out critical revisions and approved the final manuscript.

## Conflict of Interest Statement

The authors declare that the research was conducted in the absence of any commercial or financial relationships that could be construed as a potential conflict of interest.

## Footnotes

1 https://blast.ncbi.nlm.nih.gov/Blast.cgi

## References

[B1] Ali A. Parizi L. F. Ferreira B. R. Junior V. da Silva I. (2016). A revision of two distinct species of *Rhipicephalus*: *R. microplus* and *R. australis*. *Cienc. Rural* 46 1240–1248.

[B2] Altschul S. F. Gish W. Miller W. Myers E. W. Lipman D. J. (1990). Basic local alignment search tool. *J. Mol. Biol.* 215 403–410. 10.1006/jmbi.1990.9999 2231712

[B3] Apanaskevich D. A. Horak I. G. (2005). The genus *Hyalomma*. II The taxonomic status of H. (Euhyalomma) anatolicum Koch 1844 and H. (Euhyalomma) excavatum Koch 1844 with the redescription of all stages. *Acarina* 13 181–197.

[B4] Apanaskevich D. A. Horak I. G. (2008). The genus *Hyalomma* Koch, 1844: V. Re-evaluation of the taxonomic rank of taxa comprising the H. (Euhyalomma) marginatum Koch complex of species (Acari: Ixodidae) with redescription of all parasitic stages and notes on biology. *Int. J. Acarol.* 34 13–42. 10.1080/01647950808683704

[B5] Apanaskevich D. A. Horak I. G. (2009). The genus *Hyalomma* Koch, 1844. IX. Redescription of all parasitic stages of H. (Euhyalomma) impeltatum Schulze & Schlottke, 1930 and H. (E.) somalicum Tonelli Rondelli, 1935 (Acari: Ixodidae). *Syst. Parasitol.* 73 199–218. 10.1007/s11230-009-9190-x 19472079

[B6] Bakirci S. Aysul N. Eren H. Unlu A. H. Karagenc T. (2014). Diversity of ticks biting humans in Aydın province of Turkey. *Ank Univ Vet Fak Derg.* 61 93–98. 10.1501/vetfak_0000002611

[B7] Barker S. C. Walker A. R. (2014). Ticks of Australia. The species that infest domestic animals and humans. *Zootaxa.* 3816 1–144. 10.11646/zootaxa.3816.1.1 24943801

[B8] Baron S. van der Merwe N. A. Maritz-Olivier C. (2018). The genetic relationship between *R. microplus* and *R. decoloratus* ticks in South Africa and their population structure. *Mol. Phylogenet. Evol.* 129 60–69. 10.1016/j.ympev.2018.08.003 30102976

[B9] Brahma R. K. Dixit V. Sangwan A. K. Doley R. (2014). Identification and characterization of *Rhipicephalus* (*Boophilus*) *microplus* and *Haemaphysalis bispinosa* ticks (Acari: Ixodidae) of North East India by ITS2 and 16S rDNA sequences and morphological analysis. *Exp. Appl. Acarol.* 62 253–265. 10.1007/s10493-013-9732-4 23990074

[B10] Briciu V. T. Titilincu A. Ţãţulescu D. F. Cârstina D. Lefkaditis M. Mihalca A. D. (2011). First survey on hard ticks (Ixodidae) collected from humans in Romania: possible risks for tick-borne diseases. *Exp. Appl. Acarol.* 54 199–204. 10.1007/s10493-010-9418-0 21161719

[B11] Burger T. D. Shao R. Barker S. C. (2014). Phylogenetic analysis of mitochondrial genome sequences indicates that the cattle tick *Rhipicephalus* (*Boophilus*) *microplus* contains a cryptic species. *Mol. Phylogenet. Evol.* 76 241–253. 10.1016/j.ympev.2014.03.017 24685498

[B12] Cabezas-Cruz A. Allain E. Ahmad A. S. Saeed M. A. Rashid I. Ashraf K. (2019). Low genetic diversity of *Ehrlichia canis* associated with high co-infection rates in *Rhipicephalus sanguineus* (s.l.). *Parasit. Vectors* 12:12. 10.1186/s13071-018-3194-9 30616670 PMC6322249

[B13] Caetano R. L. Vizzoni V. F. Bitencourth K. Carrico C. Sato T. P. Pinto Z. T. (2017). Ultrastructural morphology and molecular analyses of tropical and temperate “species” of *Rhipicephalus sanguineus* sensu lato (Acari: Ixodidae) in Brazil. *J. Med. Entomol.* 54 1201–1212. 10.1093/jme/tjx066 28399274 PMC5850649

[B14] Cheng T. Halper B. Siebert J. Cruz-Martinez L. Chapwanya A. Kelly P. (2018). Parasites of small Indian mongoose, *Herpestes auropunctatus*, on St. Kitts, West Indies. *Parasitol. Res.* 117 989–994. 10.1007/s00436-018-5773-2 29383501 PMC5978914

[B15] Coimbra-Dores M. J. Maia-Silva M. Marques W. Oliveira A. C. Rosa F. Dias D. (2018). Phylogenetic insights on mediterranean and afrotropical *Rhipicephalus* species (Acari: Ixodida) based on mitochondrial DNA. *Exp. Appl. Acarol.* 75 107–128. 10.1007/s10493-018-0254-y 29605833

[B16] Csordas B. G. Garcia M. V. Cunha R. C. Giachetto P. F. Blecha I. M. Z. Andreotti R. (2016). New insights from molecular characterization of the tick *Rhipicephalus* (*Boophilus*) *microplus* in Brazil. *Rev. Bras. Parasitol. Vet.* 25 317–326. 10.1590/S1984-29612016053 27579530

[B17] Dantas-Torres F. (2015). Climate change, biodiversity, ticks and tick-borne diseases: the butterfly effect. *Int. J. Parasitol. Parasites Wildl.* 4 452–461. 10.1016/j.ijppaw.2015.07.001 26835253 PMC4699983

[B18] Dantas-Torres F. Latrofa M. S. Ramos R. A. N. Lia R. P. Capelli G. Parisi A. (2018). Biological compatibility between two temperate lineages of brown dog ticks. *Rhipicephalus sanguineus* (sensu lato). *Parasit. Vectors* 11:398. 10.1186/s13071-018-2941-2 29986760 PMC6038233

[B19] de la Fuente J. Antunes S. Bonnet S. Cabezas-Cruz A. Domingos A. G. Estrada-Peña A. (2017). Tick-pathogen interactions and vector competence: identification of molecular drivers for tick-borne diseases. *Front. Cell Infect. Microbiol.* 7:114. 10.3389/fcimb.2017.00114 28439499 PMC5383669

[B20] de la Fuente J. Rodriguez M. Garcia-Gari J. C. (2000). Immunological control of ticks through vaccination with *Boophilus microplus* gut antigens. *Ann. N Y Acad Sci.* 916 617–621. 10.1111/j.1749-6632.2000.tb05347.x 11193686

[B21] Estrada-Peña A. (2008). Climate, niche, ticks, and models: what they are and how we should interpret them. *Parasitol. Res.* 103 87–95. 10.1007/s00436-008-1056-7 19030890

[B22] Estrada-Peña A. Gray J. S. Kahl O. Lane R. S. Nijhoff A. M. (2013). Research on the ecology of ticks and tick-borne pathogens—methodological principles and caveats. *Front. Cell Infect. Microbiol.* 3:29. 10.3389/fcimb.2013.00029 23964348 PMC3737478

[B23] Estrada-Peña A. Jongejan F. (1999). Ticks feeding on humans: a review of records on human-biting Ixodoidea with special reference to pathogen transmission. *Exp. Appl. Acarol.* 23 685–715. 10581710 10.1023/a:1006241108739

[B24] Estrada-Peña A. Venzal J. M. Nava S. Mangold A. Guglielmone A. A. Labruna M. B. (2012). Reinstatement of *Rhipicephalus* (*Boophilus*) australis (Acari: Ixodidae) with redescription of the adult and larval stages. *J. Med. Entomol.* 49 794–802. 10.1603/me11223 22897039

[B25] Fernández-Soto P. Pérez-Sánchez R. Álamo-Sanz R. Encinas-Grandes A. (2006). Spotted fever group rickettsiae in ticks feeding on humans in northwestern Spain: is *Rickettsia conorii* vanishing? *Ann. N Y Acad Sci.* 1078 331–333. 10.1196/annals.1374.063 17114733

[B26] García-García J. C. Gonzalez I. L. González D. M. Valdés M. Méndez L. Lamberti J. (1999). Sequence variations in the *Boophilus microplus* Bm86 locus and implications for immunoprotection in cattle vaccinated with this antigen. *Exp. Appl. Acarol.* 23 883–895. 10668863 10.1023/a:1006270615158

[B27] Ghosh H. S. Misra K. K. (2012). Scanning electron microscope study of a snake tick, *Amblyomma gervaisi* (Acari: Ixodidae). *J. Parasit. Dis.* 36 239–250. 10.1007/s12639-012-0117-0 24082536 PMC3427664

[B28] Gondard M. Cabezas-Cruz A. Charles R. A. Vayssier-Taussat M. Albina E. Moutailler S. (2017). Ticks and tick-borne pathogens of the Caribbean: current understanding and future directions for more comprehensive surveillance. *Front. Cell Infect. Microbiol.* 7:490. 10.3389/fcimb.2017.00490 29238699 PMC5713125

[B29] Gratz N. G. (1999). Emerging and resurging vector-borne diseases. *Annu. Rev. Entomol.* 44 51–75. 10.1146/annurev.ento.44.1.51 9990716

[B30] Guglielmone A. A. Robbins R. G. (2018). *Hard Ticks (Acari: Ixodida: Ixodidae) Parasitizing Humans*, (New York, NY: Springer), 314.

[B31] Guglielmone A. A. Robbins R. G. Apanaskevich D. A. Petney T. N. Estrada-Peña A. Horak I. G. (2014). *The hard ticks of the world.* Dordrecht: Springer.

[B32] Hall T. Biosciences I. Carlsbad C. (2011). BioEdit: an important software for molecular biology. *GERF Bull. Biosci.* 2 60–61. 10.1016/j.compbiolchem.2019.02.002 30769268

[B33] Hanson B. A. Frank P. A. Mertins J. W. Corn J. L. (2007). Tick paralysis of a snake caused by *Amblyomma rotundatum* (Acari: Ixodidae). *J. Med. Entomol.* 44 155–157. 10.1093/jmedent/41.5.155 17294934

[B34] Hoogstraal H. (1970). Identity, distribution, and hosts of *Haemaphysalis* (*Rhipistoma*) *indica* Warburton (resurrected) (Ixodoidea: Ixodidae), a carnivore parasite of the Indian subregion. *J. Parasitol.* 56 1013–1022.

[B35] Hoogstraal H. Varma M. G. R. (1962). Haemaphysalis cornupunctata sp. n. and *H. kashmirensis* sp. n. from Kashmir, with Notes on H. sundrai Sharif and H. sewelli Sharif of India and Pakistan (Ixodoidea, Ixodidae). *J. Parasitol.* 48 185–194. 13908757

[B36] Hoogstraal H. Wassef H. Y. (1977). Haemaphysalis (Ornithophysalis) kadarsani sp. n. (Ixodoidea: Ixodidae), a rodent parasite of virgin lowland forests in Sulawesi (Celebes). *J. Parasitol.* 63 1103–1109.

[B37] Horak I. G. Chaparro F. Beaucournu J. C. Louw J. P. (1999). Parasites of domestic and wild animals in South Africa. XXXVI. Arthropod parasites of yellow mongooses, *Cynictis penicillata* (G. Cuvier, 1829). *Onderstepoort J. Vet. Res.* 66 33–38. 10396760

[B38] Horak I. G. Fourie L. J. Braack L. E. O. (2005). Small mammals as hosts of immature ixodid ticks. *Onderstepoort J. Vet. Res.* 72 255–261. 16300195 10.4102/ojvr.v72i3.204

[B39] Jabbar A. Abbas T. Saddiqi H. A. Qamar M. F. Gasser R. B. (2015). Tick-borne diseases of bovines in Pakistan: major scope for future research and improved control. *Parasit. Vectors* 8:283. 10.1186/s13071-015-0894-2 25994588 PMC4443554

[B40] Jones K. E. Patel N. G. Levy M. A. Storeygard A. Balk D. Gittleman J. L. (2008). Global trends in emerging infectious diseases. *Nature* 451 990–993. 10.1038/nature06536 18288193 PMC5960580

[B41] Jore S. Vanwambeke S. O. Viljugrein H. Isaksen K. Kristoffersen A. B. Woldehiwet Z. (2014). Climate and environmental change drives *Ixodes ricinus* geographical expansion at the northern range margin. *Parasit. Vectors* 7:11. 10.1186/1756-3305-7-11 24401487 PMC3895670

[B42] Kaiser M. N. Hoogstraal H. (1964). The *Hyalomma* ticks (Ixodoidea, Ixodidae) of Pakistan, India, and Ceylon, with keys to subgenera and species. *Acarologia* 6 257–286.

[B43] Kar S. Yilmazer N. Akyildiz G. Gargili A. (2017). The human infesting ticks in the city of Istanbul and its vicinity with reference to a new species for Turkey. *Syst. Appl. Acarol.* 22 2245–2256.

[B44] Karim S. Budachetri K. Mukherjee N. Williams J. Kausar A. Hassan M. J. (2017). A study of ticks and tick-borne livestock pathogens in Pakistan. *PLoS Negl. Trop Dis.* 11:e0005681. 10.1371/journal.pntd.0005681 28650978 PMC5501686

[B45] Kumar S. Stecher G. Li M. Knyaz C. Tamura K. (2018). MEGA X: molecular evolutionary genetics analysis across computing platforms. *Mol. Biol. Evol.* 35 1547–1549. 10.1093/molbev/msy096 29722887 PMC5967553

[B46] Labruna M. B. Jorge R. S. Sana D. A. Jácomo A. T. Kashivakura C. K. Furtado M. M. (2005). Ticks (Acari: Ixodida) on wild carnivores in Brazil. *Exp. Appl. Acarol.* 36 149–163. 16082932 10.1007/s10493-005-2563-1

[B47] Labruna M. B. McBride J. W. Bouyer D. H. Camargo L. M. A. Camargo E. P. Walker D. H. (2004). Molecular evidence for a spotted fever group *Rickettsia* species in the tick *Amblyomma longirostre* in Brazil. *J. Med. Entomol.* 41 533–537. 15185961 10.1603/0022-2585-41.3.533

[B48] Labruna M. B. Naranjo V. Mangold A. J. Thompson C. Estrada-Peña A. Guglielmone A. A. (2009). Allopatric speciation in ticks: genetic and reproductive divergence between geographic strains of *Rhipicephalus* (*Boophilus*) microplus. *BMC Evol. Biol.* 9:46. 10.1186/1471-2148-9-46 19243585 PMC2656471

[B49] Leger E. Vourc’h G. Vial L. Chevillon C. McCoy K. D. (2013). Changing distributions of ticks: causes and consequences. *Exp. Appl. Acarol.* 59 219–244. 10.1007/s10493-012-9615-0 23015121

[B50] Lempereur L. Geysen D. Madder M. (2010). Development and validation of a PCR–RFLP test to identify African *Rhipicephalus* (*Boophilus*) ticks. *Acta Trop.* 114 55–58. 10.1016/j.actatropica.2010.01.004 20080073

[B51] Lew-Tabor A. E. Ali A. Rehman G. Rocha-Garcia G. Zangirolamo A. F. Malardo T. (2017). Cattle Tick *Rhipicephalus microplus*-host interface: a review of resistant and susceptible host responses. *Front. Cell Infect Microbiol.* 7:506. 10.3389/fcimb.2017.00506 29322033 PMC5732177

[B52] Lew-Tabor A. E. Valle M. R. (2016). A review of reverse vaccinology approaches for the development of vaccines against ticks and tick borne diseases. *Ticks Tick Borne Dis.* 7 573–585. 10.1016/j.ttbdis.2015.12.012 26723274

[B53] Low V. L. Tay S. T. Kho K. L. Koh F. X. Tan T. K. Lim Y. A. L. (2015). Molecular characterisation of the tick *Rhipicephalus microplus* in Malaysia: new insights into the cryptic diversity and distinct genetic assemblages throughout the world. *Parasit. Vectors* 8:341. 10.1186/s13071-015-0956-5 26104478 PMC4482097

[B54] Lv J. Wu S. Zhang Y. Zhang T. Feng C. Jia G. (2014). Development of a DNA barcoding system for the Ixodida (Acari: Ixodida). *Mitochondrial DNA* 25 142–149. 10.3109/19401736.2013.792052 23631370

[B55] Madder M. Horak I. Stoltsz H. (2014). *Tick Identification.* Pretoria: Faculty of veterinary Science University of Pretoria, 58.

[B56] Manan A. Khan Z. Ahmad B. (2007). Prevalence and identification of ixodid tick genera in frontier region Peshawar. *J. Agric. Biol. Sci.* 37 173–176.

[B57] Marcus L. C. (1981). *Veterinary Biology and Medicine of Captive Amphibians and Reptiles.* Philadelphia, PA: Lea and Febiger.

[B58] Nowak-Chmura M. (2012). Teratological changes in tick morphology in ticks feeding on exotic reptiles. *J. Nat. Hist.* 46 911–921. 10.1080/00222933.2011.651635

[B59] Okino T. Ushirogawa H. Matoba K. Hatsushika R. (2010). Bibliographical studies on human cases of hard tick (Acarina: Ixodidae) bites in Japan (5) cases of *Ixodes acutitarsus* and *I. turdus* infestation. *Kawasaki Med. J.* 36 115–120.

[B60] Paddock C. D. Childs J. E. (2003). *Ehrlichia chaffeensis*: a prototypical emerging pathogen. *Clin. Microbiol. Rev.* 16 37–64. 10.1128/cmr.16.1.37-64.2003 12525424 PMC145301

[B61] Papa A. Karabaxoglou D. Kansouzidou A. (2011). Acute West Nile virus neuroinvasive infections: cross-reactivity with dengue virus and tick-borne encephalitis virus. *J. Med. Virol.* 83 1861–1865. 10.1002/jmv.22180 21837806

[B62] Parola P. Raoult D. (2001). Ticks and tick-borne bacterial diseases in humans: an emerging infectious threat. *Clin. Infect. Dis.* 32 897–928. 10.1086/319347 11247714

[B63] Rehman A. Nijhof A. M. Sauter-Louis C. Schauer B. Staubach C. Conraths F. J. (2017). Distribution of ticks infesting ruminants and risk factors associated with high tick prevalence in livestock farms in the semi-arid and arid agro-ecological zones of Pakistan. *Parasit. Vectors* 10:190. 10.1186/s13071-017-2138-0 28420420 PMC5395890

[B64] Robertson R. G. Wisseman C. L. Traub R. (1970). Tick-Borne Rickettsiae of the Spotted Fever Group in West Pakistan: I. isolation of strains from ticks in different habitats. *Am J Epidemiol.* 92 382–394. 10.1093/oxfordjournals.aje.a121221 5536972

[B65] Roy B. C. Krücken J. Ahmed J. S. Majumder S. Baumann M. P. Clausen P. H. (2018). Molecular identification of tick-borne pathogens infecting cattle in Mymensingh district of Bangladesh reveals emerging species of Anaplasma and Babesia. *Transbound. Emerg. Dis.* 65 231–242. 10.1111/tbed.12745 29119682

[B66] Santos-Silva M. M. Beati L. Santos A. S. De Sousa R. Núncio M. S. Melo P. (2011). The hard-tick fauna of mainland Portugal (Acari: Ixodidae): an update on geographical distribution and known associations with hosts and pathogens. *Exp. Appl. Acarol.* 55 85–121. 10.1007/s10493-011-9440-x 21452063

[B67] Schorderet-Weber S. Noack S. Selzer P. M. Kaminsky R. (2017). Blocking transmission of vector-borne diseases. *Int. J. Parasitol. Drugs Drug Resist.* 7 90–109. 10.1016/j.ijpddr.2017.01.004 28189117 PMC5302141

[B68] Serra-Freire N. M. (2010). Occurrence of ticks (Acari: Ixodidae) on human hosts, in three municipalities in the State of Pará, Brazil. *Rev. Bras. Parasitol. Vet.* 19 141–147. 10.1590/s1984-29612010000300003 20943016

[B69] Sherrard-Smith E. Chadwick E. Cable J. (2012). Abiotic and biotic factors associated with tick population dynamics on a mammalian host: *Ixodes hexagonus* infesting otters. *Lutra. PloS One* 7:e47131. 10.1371/journal.pone.0047131 23071736 PMC3465257

[B70] Stenos J. Graves S. R. Unsworth N. B. (2005). A highly sensitive and specific real-time PCR assay for the detection of spotted fever and typhus group *Rickettsiae*. *Am. J. Trop Med. Hyg.* 73 1083–1085. 10.4269/ajtmh.2005.73.1083 16354816

[B71] Stephen S. Achyutha R. (1979). Q fever in South Kanara district: natural occurrence of *Coxiella burneti* in the tick (*Aponomma gervaisi*) preliminary report. *Indian J. Med. Res.* 69 244–246. 429027

[B72] Tadesse F. Abadfaji G. Girma S. Jibat T. (2012). Identification of tick species and their preferred site on cattles body in and around Mizan Teferi, Southwestern Ethiopia. *Vet. Med. Anim. Health* 4 1–5.

[B73] Tamura K. Stecher G. Peterson D. Filipski A. Kumar S. (2013). MEGA 6 molecular evolutionary genetics analysis version 6.0. *Mol. Biol. Evol.* 30 2725–2729. 10.1093/molbev/mst197 24132122 PMC3840312

[B74] Tenderio J. (1953). Alguns dados sobre as stirpes de *Coxiella burneti* isoladas na Guine Portugusea. *Bol. Cult. Guine Port.* 8 69–87.

[B75] Walker A. R. Bouattour A. Camicas J. L. Estrada-Peña A. Horak I. G. Latif A. A. (2003). *Ticks of Domestic Animals in Africa: A Guide to Identification of Species.* Edinburgh: Bioscience Reports.

[B76] Yu Z. Wang H. Wang T. Sun W. Yang X. Liu J. (2015). Tick-borne pathogens and the vector potential of ticks in China. *Parasit. Vectors* 8:24. 10.1186/s13071-014-0628-x 25586007 PMC4300027

